# Extraction Optimization and Qualitative/Quantitative Determination of Bioactive Abietane-Type Diterpenes from Three *Salvia* Species (Common Sage, Greek Sage and Rosemary) by ^1^H-qNMR

**DOI:** 10.3390/molecules29030625

**Published:** 2024-01-28

**Authors:** Panagiotis Kallimanis, Prokopios Magiatis, Angeliki Panagiotopoulou, Kostas Ioannidis, Ioanna Chinou

**Affiliations:** 1 Laboratory of Pharmacognosy and Chemistry of Natural Products, Department of Pharmacy, National and Kapodistrian University of Athens, 15771 Zografou, Greece; pgkallimanis@gmail.com; 2 Institute of Biosciences & Applications, National Centre for Scientific Research “Demokritos”, 15310 Agia Paraskevi Attikis, Greece; apanagio@bio.demokritos.gr; 3 Laboratory of Sylviculture, Forest Genetics and Biotechnology, Institute of Mediterranean and Forest Ecosystems, Hellenic Agricultural Organization “Demeter”, Ilissia, 11528 Athens, Greece; ioko@fria.gr

**Keywords:** *Salvia officinalis*, *Salvia fruticosa*, *Salvia rosmarinus*, infusions, decoctions, oleolites, tinctures, carnosic acid, carnosol, rosmanol

## Abstract

The objective of this study was the optimization of the extraction process and the qualitative and quantitative determination of the bioactive metabolites: 12-*O*-methylcarnosic acid (12MCA), carnosic acid (CA), carnosol (CS), 7-*O*-methyl-*epi*-rosmanol (7MER) and rosmanol (RO) in infusions, decoctions, turbulent flow extracts, tinctures and oleolites from three *Salvia* species: *Salvia officinalis* L. (common sage, *SO*), *Salvia fruticosa* Mill. (Greek sage, *SF*) and *Salvia rosmarinus* Spenn (syn *Rosmarinus officinalis* L.) (rosemary, *SR*), using Quantitative Proton Nuclear Magnetic Resonance Spectroscopy (^1^H-qNMR). Regarding the aqueous extracts, decoctions appeared to be richer sources of the studied metabolites than infusions among the three plants. For *SR*, the turbulent flow extraction under heating was the most efficient one. The optimum time for the preparation of decoctions was found to be 5 min for *SF* and *SO* and 15 min for *SR*. It is noteworthy that *SR* tinctures were not stable in time due to decomposition of the abietane-type diterpenes CA and CS because of the polar solvent used for their preparation. Contrary to this finding, the oleolites of *SR* appeared to be very stable. Olive oil as a solvent for extraction was very protective for the contained abietane-type diterpenes. A preliminary stability study on the effect of the storage time of the *SF* on the abietane-type diterpenes content showed that the total quantity of abietanes decreased by 16.51% and 40.79% after 12 and 36 months, respectively. The results of this investigation also demonstrated that ^1^H-qNMR is very useful for the analysis of sensitive metabolites, like abietane-type diterpenes, that can be influenced by solvents used in chromatographic analysis.

## 1. Introduction

Lamiaceae (syn = Labiatae) is the sixth largest plant family, with >200 genera and >7000 species widespread and easily cultivated worldwide. Most of them have aromatic and therapeutic properties which make them an extremely important part of the food, cosmetic and pharmaceutical industries [1]. The plants selected and studied in the current work were the common sage (*Salvia officinalis* L.), the Greek *sage* (*Salvia fruticosa* Mill.) and *rosemary* (*Salvia rosmarinus* Spenn syn = *Rosmarinus officinalis* L.). The fresh or dried leaves of these globally recognized plants are commonly utilized as culinary spices, in traditional medicine, phytotherapy and even as food preservatives. Moreover, these plants are also accepted in conventional medicine according to the World Health Organization (WHO) as well as the European Medicines Agency (EMA), due to their pharmacological properties towards the relief of symptoms of different diseases based on their long traditional uses and confirmed therapeutic effects [2,3,4,5,6,7,8,9,10,11].

Rosemary (*SR*), common sage (*SO*) and Greek sage (*SF*) are recognized for their strong antioxidant activity and pharmacological properties—mostly attributed to the presence of phenolic compounds and abietane-type diterpenes. All three plants contain valuable bioactive metabolites: carnosic acid (CA), carnosol (CS), 12-*O*-methylcarnosic acid (12MCA), rosmanol (RO) and 7-*O*-methyl-*epi*-rosmanol (7MER), which are highly interesting due to their anti-inflammatory, anti-oxidant, anti-tumor, anti-HIV, anti-microbial, anti-Alzheimer, anti-adipogenic and neuroprotective properties [12]. All these metabolites have been identified uniquely in specific plants of the Lamiaceae family [13,14]. Indeed, CA accounts for about 30% of phytochemical studies and is the most studied bioactive metabolite of *SR*, followed by CS with 17%, while both rosmarinic acid and ursolic acid add up to 18% of research studies [3].

*SR*, *SO* and *SF* leaves have been used in folk medicine widely and later in phytotherapy as approved medicinal products, mostly in the form of herbal teas [8,10]. These plant preparations include infusions, decoctions, macerations, tinctures and/or oleolites for oral use. Traditional forms of application for medicinal purposes include herbal preparations in the form of *infusum* (infusion obtained by pouring boiling water over the plant material and allowing it to steep for a defined period, typically measured in minutes), decoctum (decoction obtained by pouring cold water on the herbal substance, bringing it to a boil and allowing it to simmer for a defined period of time) and maceratum (macerate obtained by soaking the herbal substance in solvent at room temperature for a defined period of time). When the maceration is performed with gentle heating at a temperature higher than room temperature, but not to boiling, the process is termed “digestion” [15,16].

The word oleolites refers to the Greek word *ελαιόλυτα* that means “dissolved in oil”. Oleolite is an effective extraction method using an edible oil as solvent at room temperature for several days or assisting the extraction capacity with higher temperature. The most common edible oil used in traditional medicine in Greece is extra virgin olive oil (*Olea europaea* L.), which has also been used in our experiments [17].

There are a lot of data regarding procedures for final herbal preparations containing rosemary or sage [8,10]; however, they are not based in objective criteria and they lack adequate specific information. In many cases of plant extracts, it is unclear or not well-established whether it is preferable to use a decoction or an infusion for each specific plant. In addition, it is not known which would be the optimum time for the plant’s extraction, the drug/solvent ratio, the extraction solvent and the potential stability of the herbal preparations.

In the framework of our studies on Mediterranean medicinal and/or aromatic plants, we recently reported that the methanolic extract of *SR* at 100 ppm antagonized the activity of the well-known environmental pollutant TCDD (2,3,7,8-tetrachlorodibenzo-*p*-*dioxin*) and of ligands of natural origin, in this case *Malassezia* metabolites, as FICZ (6-formylindolo [3,2-*b*]carbazole), PZ (pityriazepin) and IND (indirubin) on AhR (aryl hydrocarbon receptor or dioxin receptor), by 97.65%, 44.96%, 82.09% and 90.09%, respectively, in human keratinocytes. Moreover, the compounds CA, CS and 7MER, isolated from the leaves of *SR*, were able to antagonize in vitro the action of TCDD on AhR by 81.93%, 78.41% and 66.59%, respectively. The antagonist activity exhibited by the rosemary extract and their isolated metabolites was dose-dependent. The discovery of natural agents that act as competitors of AhR seems of great importance as they could be used to prevent or treat established dioxin toxicity. These substances could be used for the treatment of chloracne as well as for both the prevention and treatment of skin cancer or inflammatory skin diseases [18].

In the present work, qualitative and quantitative analyses of different types of aqueous extracts (infusions, decoctions, turbulent flow extractions), tinctures and oleolites of *SR*, *SO* and *SF* leaves were performed tracing selected abietane-type diterpenes (CA, CS, 7MER, 12MCA, RO), without any separation steps, using quantitative proton Nuclear Magnetic Resonance Spectroscopy (^1^H-qNMR). The isolation procedure and full structural determination of the forementioned metabolites have been fully described recently by our scientific team [17,18]. Apart from conventional solvent extraction methods, currently, solvent microextrations have come forward as being effective modern sample preparation approaches [19]. The scope of the current study was to present the qualitative and quantitative analyses of all three herbs as well as to validate and compare different extraction methods and parameters (extraction solvents, drug/solvent ratio, etc.) towards the optimization of different abietane-type diterpenes’ extraction using qNMR.

qNMR spectroscopy is superior to traditional quantitation methods as there is no need for an analyte calibration standard and only an inexpensive internal standard is required, while high selectivity can be achieved under appropriate acquisition conditions and more than one analyte can be determined at the same time. These result in reduced measuring time and thus avoid sample deterioration.

The aim of the current investigation was to design, optimize and validate the extraction method for the targeted bioactive compounds of abietane-type diterpenes present in different preparation methods. The samples were prepared as infusions and decoctions (prepared for 2, 5, 10 and 15 min for each), turbulent flow extractions (at room temperature and at 100 °C), tinctures (in two different ethanol volumes of 20% and 70%) and as oleolites (prepared at room temperature and at 65 °C)

**Figure 1:**
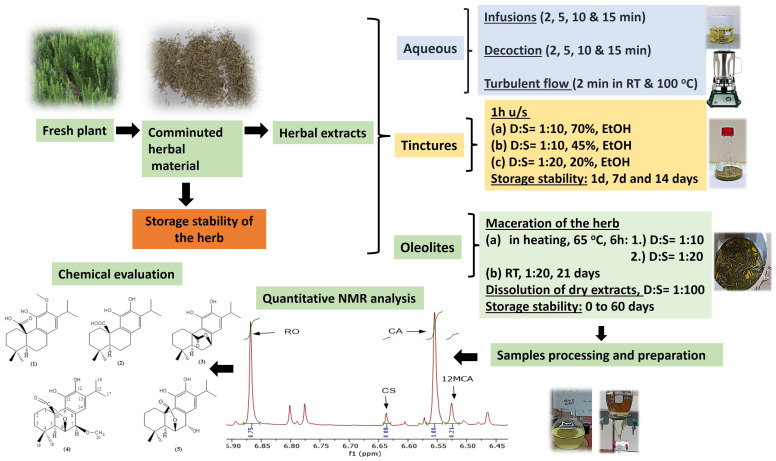
Schematic representation of experimental design.

Moreover, a study about the effect of the storage time of the herbs prior to the extraction on the qualitative and quantitative characteristics towards abietane-type diterpenes content has been performed. An overview of the experimental design is presented in Figure 1.

## 2. Results

### 2.1. Quantitation of the Metabolites

For the quantitation of the studied metabolites, all ^1^H- NMR signals were assigned.

**Figure 2:**
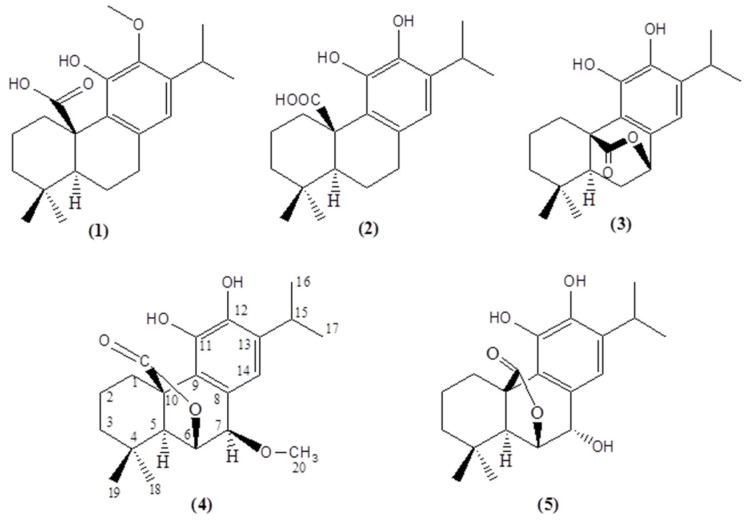
The structures of metabolites isolated and quantitated: 12-*O*-methylcarnosic acid (12MCA) (**1**), carnosic acid (CA) (**2**), carnosol (CS) (**3**), 7-*O*-methyl-*epi*-rosmanol (7MER) (**4**) and rosmanol (RO) (**5**).

Simple (s) peaks integrating for one proton were observed at 6.56 ppm (H-14) of carnosic acid, at 6.64 ppm (H-14) of carnosol, at 6.53 ppm (H-14) of 12-*O*-methyl-carnosic acid, at 6.79 ppm (H-14) of 7-methyl-epirosmanol and at 6.86 (H-14) of rosmanol, respectively. The structures of the studied metabolites are presented in Figure 2.

The limit of detection (LOD) was set to the value (mg/mL) corresponding to three times the noise value (LOD = 3 × noise signal). The limit of quantification (LOQ) was set as the value (mg/mL) corresponding to 10 times the noise value (LOQ = 10 × noise signal). For quantitative analysis, a calibration curve for isolated abietane-type diterpenes was constructed based on selected non-overlapping ^1^H-NMR signals (Table 1).

### 2.2. Aqueous Extracts

**Figure 3:**
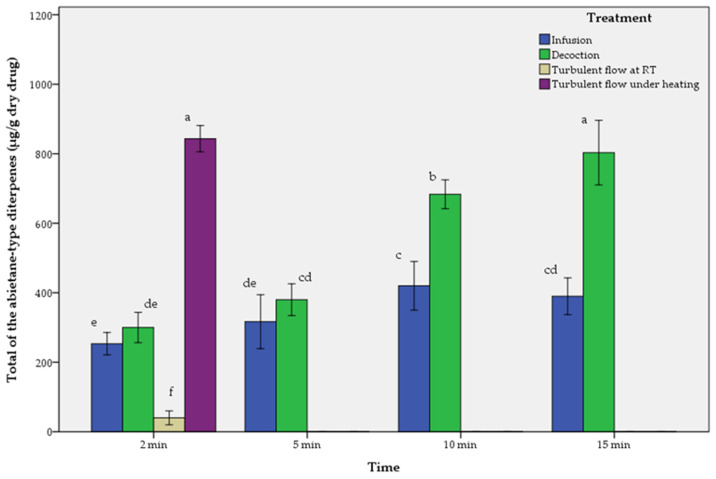
Comparison of infusions (I), decoctions (D) and turbulent flow extractions (T) of rosemary (*SR*) depending on time. Means followed by the same letter do not differ statistically at *p* = 0.05. T-bars represent ±1 sd.

**Figure 4:**
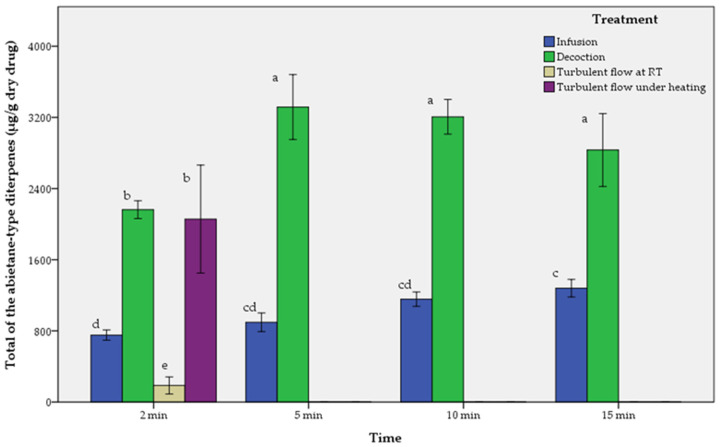
Comparison of infusions (I), decoctions (D) and turbulent flow extractions (T) of Greek sage (*SF*) depending on time. Means followed by the same letter do not differ statistically at *p* = 0.05. T-bars represent ±1 sd.

**Figure 5:**
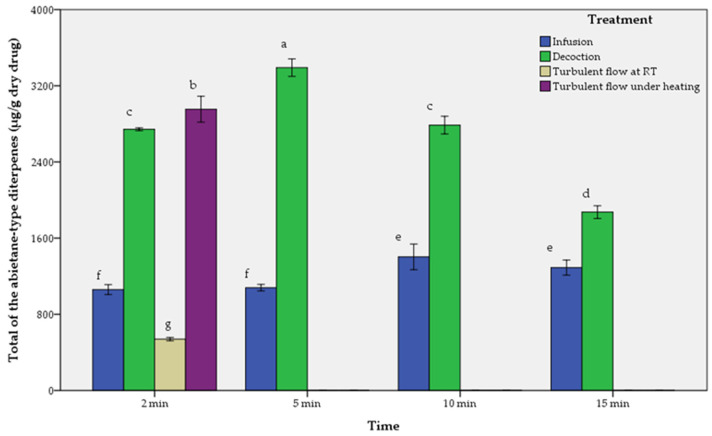
Comparison of infusions (I), decoctions (D) and turbulent flow extractions (T) of common sage (*SO*) depending on time. Means followed by the same letter do not differ statistically at *p* = 0.05. T-bars represent ±1 sd.

The results of aqueous extracts are presented in Table 2, Table 3 and Table 4 and in Figure 3, Figure 4 and Figure 5.

As described in Table 2, Table 3 and Table 4, and Figure 3, Figure 4 and Figure 5, the comparison of performance between infusions, decoctions and turbulent flow extractions regarding all the tested abietane-type diterpenes is in descending order as follows:

- For rosemary (*SR*):

Turbulent flow extraction under heating > Decoction_15 min > Decoction_10 min > Infusion_10 min > Infusion_15 min > Decoction_5 min > Infusion_5 min > Decoction_2 min > Infusion_2 min > Turbulent flow extraction at room temperature. The maximum value of CA, CS was in turbulent flow extraction under heating, while for RO in infusion_10 min.

- For Greek sage (*SF*):

Decoction_5 min > Decoction_10 min > Decoction_15 min > Decoction_2 min > Turbulent flow extraction under heating > Infusion_15 min > Infusion_10 min > Infusion_5 min > Infusion_2 min > Turbulent flow extraction at room temperature. The maximum values of CA and CS were at 5 min of decoction, while for RO at 10 min of decoction.

- For common sage (*SO*):

Decoction_5 min > Turbulent flow extraction under heating > Decoction_10 min > Decoction_2 min > Decoction_15 min > Infusion_10 min > Infusion_15 min > Infusion_5 min > Infusion_2 min. The maximum values of CA and CS were at turbulent flow extraction under heating, while for RO at 10 min of decoction.

### 2.3. Tinctures

**Figure 6:**
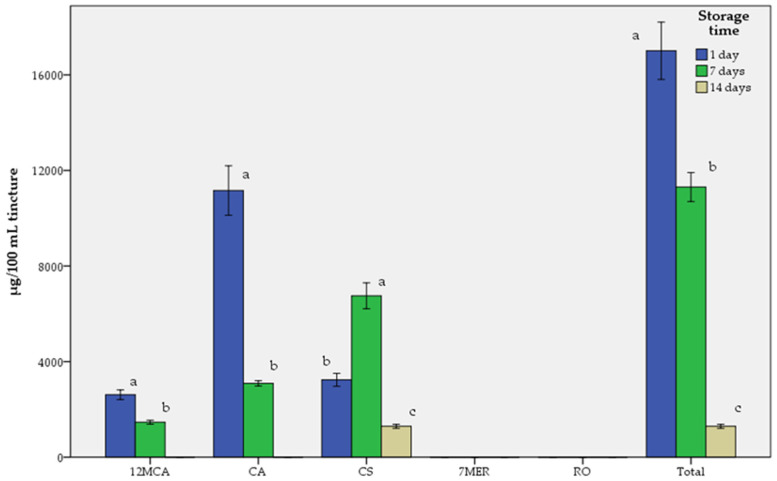
Tinctures stability and storage time of abietane-type diterpenes of rosemary (*SR*). Means followed by the same letter for each substance, at different storage times, do not exhibit statistically significant differences at *p* = 0.05. T-bars represent ±1 sd.

The results of tinctures are presented in Table 5 and Table 6 and in Figure 6.

As described in Table 5 at the same ratio of drug/solvent, the increase in ethanol percentage leads to a higher total of abietane-type diterpenes.

The results that appear in Table 6 and Figure 6 show that at the first day of storage, the amounts of abietane-type diterpenes in descending order were CA > CS > 12MCA > RO, while after 7 days of storage, the order was CS > CA > 12MCA > RO, due to the transformation of CA in CS, and after 14 days of storage, only CS was quantified, while 12MCA, CA and RO were in traces. 7MER was not identified in any experiment. The decrease in CA and 12MCA was directly proportional to the increase in the storage time of the tincture and it was observed that CA had the largest decrease. The total reduction in tested abietane-type diterpenes at 14 days was measured at 92.42%.

### 2.4. Oleolites

The results of oleolites are presented in Table 7 and Table 8.

An experiment was devised to explore how the stability of the tested substances in olive oil relates to their storage time at room temperature. The oleolite produced through maceration (21 days of maceration in protection from light, drug: olive oil ratio = 1:20 *w*/*w*, at room temperature) was left undisturbed in an opaque bottle for 2 months, stored in a light-protected environment. The oleolite was analyzed at the beginning and at the end of the storage period and the results are presented in Table 9.

An additional experiment was conducted to compare the extractive performance of olive oil, ethanol and methanol under the same conditions (drug to solvent ratio 1:10 *w*/*v,* 60 min, ultrasonic bath). The results are presented in Table 10.

### 2.5. Study of the Effect of the Storage Time of the Drug on the Qualitative and Quantitative Characteristics of Abietane-Type Diterpenes

The results are presented in Table 11.

### 2.6. Study on 7MER

7MER was not identified in any experiment. To analyze this fact, we macerated the dry leaves of ***SR*** in methanol for four different times and the results are shown in Table 12.

## 3. Discussion

*SF*, *SO* and *SR* are plants well known for their beneficial actions on human health since antiquity. In most cases, these herbs are used as infusions, decoctions, tinctures and oleolites in folk medicine, in phytotherapy and in the food industry as natural, non-toxic preservatives. After carefully reviewing the available monographs from EMA [8,10] and focusing on the abietane-type diterpenes, which according to the literature of recent years are considered the most active substances of the aforementioned plants [3,4], we found that the impact of the extraction method (decoction, infusion, tincture, oleolite) and the extraction parameters (drug solvent ratio, time of extraction) on the chemical profile as well as the stability of each extract had never been systematically studied.

Based on the obtained results, we can confidently estimate the optimal times for the extraction of the total of the tested abietane-type diterpenes. Infusion of dried rosemary leaves (*SR*) requires 10–15 min, while infusion of dried Greek sage leaves (*SF*) needs 15 min. In case that we would like to obtain the largest amount of CA and CS from our infusion, then the infusion should be done at 15 min. Infusion of dried common sage leaves (*SO*) also requires 10–15 min (largest amount of CA and CS at 15 min). Similarly, the decoction of dried rosemary leaves (*SR*) requires 15 min, but the decoction of the dried Greek sage leaves (*SF*) needs only 5 min (largest amount of CA and CS at these times, respectively), as does the decoction of the dried common sage leaves (*SO*). Regarding the efficiency in obtaining the aqueous extracts (turbulent flow extraction-infusion-decoction) of the aforementioned plants, we have found that for rosemary (*SR*), the most efficient extraction process is turbulent flow extraction under heating (TFE ≥ decoction > infusion), while for Greek sage (*SF*) and common sage (*SO*), it is decoction (decoction ≥ TFE > infusion). Generally, for all plants studied, it was proven that decoction was more efficient than infusion in the same process time. Additionally, in the turbulent extraction at ambient temperature, the total of the studied diterpenes was lower than that found in infusions and decoctions at any extraction time.

According to the results of aqueous extracts (the turbulent flow extractions, infusions and decoctions) of rosemary (*SR*), Greek sage (*SF*) and common sage (*SO*), it appears that the respective preparations of common sage (*SO*) are richer than those of rosemary (*SR*) and Greek sage (*SF*) in both the total abietane-type diterpenes examined as well as in the individually measured substances CA, CS and 12MCA, in all conditions of the experiments.

Turbulent flow extraction is a rapid extraction method that can combine rigorous mixing of the plant material with the solvent (e.g., cold or hot water) with the simultaneous reduction in the size of the plant material particles [20]. Turbulent flow extraction with heating was found to be more efficient across all three examined plants compared to the processes taking place at ambient temperature. Additionally, the yield from turbulent flow extraction with heating exceeded that of infusions from all the tested plants and at all infusion times. The value of turbulent flow is also shown by the fact that both experiments, infusions and turbulent flow extraction in heating were performed at the same temperature. To our knowledge, in this study, we present for the first time the results of the turbulent flow extraction, in the examined temperatures, for the aforementioned plants.

Due to the known sensitivity of CA and CS to polar solvents, such as water and ethanol [21,22], an experiment has been performed to estimate the rate of degradation of the tested substances in an hydroalcoholic solution (tincture) as a result of storage time for a period of up to 14 days (t1 = 1 day, t2 = 7 days, t3 = 14 days). The stability experiments indicate that tinctures, as a water–alcohol solution, might not be a suitable pharmaceutical form for rosemary (*SR*) due to the rapid degradation of CA, CS and 12MCA over time. Indeed, it appears that in just 14 days the main group of the active components showed a quantitatively significant reduction and a possible consequent decrease in their bioactivity. However, in case we want to make a dry extract from the leaves of *SR* using an ethanolic solution, we should bear in mind that the yield of the extraction increases by going from 45 to 70% alcohol. Therefore, to prepare a dry extract from the dried leaves of *SR* using an ethanolic solution, the solvent should be 70% alcohol. It is also crucial to limit the contact time of the drug with the solvent during maceration to 24 h.

Regarding the oleolites (oil solutions) of rosemary (*SR*) and Greek sage (*SF*):

(i) CA, CS and 12MCA are extracted (starting from dried leaves) and dissolved (starting with dry extract) in olive oil.
(ii) In the experiment investigating the stability of the examined substances in olive oil as a function of storage time (2 months), which was carried out at room temperature and in a place protected from light, it was found that the total amount of the tested abietane-type diterpenes decreased only by 4.31%. Hence, CA, CS and 12MCA are quite stable in extra virgin olive oil, which behaves as a lipophilic, non-polar solvent. This is a very important finding considering the easy and fast conversion of CA, CS and 12MCA (especially CA, CS) in polar solvents such as water, ethanol and methanol. By using olive oil for the preparation of an oleolite containing the CA, CS and 12MCA, we can have a pharmaceutical form where the compounds of interest are very stable, at least for the first 2 months after its preparation, as was shown by the experimental results described in Section 2.4.

The results of the oil extracts of rosemary (*SR*) leaves by maceration at room temperature and with heating suggest that:

- With the same drug/solvent ratio maceration in oil at room temperature for 21 days in the absence of light, the extraction was more efficient than by heating at 65 °C for 6 h with regard to all of the tested abietane-type diterpenes.
- With the same method, maceration in oil at 65° for 6 h, but with different proportions of drug solvent 1:10 and 1:20 *w*/*w*, the ratio 1:10 *w*/*w* was more efficient.

Overall, the most efficient method among those tested is heating at 65 °C for 6 h and the most suitable ratio of solvent drug is the ratio of 1:10 *w*/*w*.

According to the results of the oleolites made by dissolution of dry methanolic extracts of *SR* and *SF* in extra virgin olive oil, it was shown that:

- In rosemary (*SR*)
  (a) CA was detected as 12.16% of the amount present in the methanolic extract.
  (b) CS and 12MCA were detected in the oleolite.
  (c) The total of the abietane-type diterpenes tested in the oil was 7.47% of the amount present in the methanolic extract.
- In Greek sage (*SF*)
  (a) CA was detected as 12.57% of the amount present in the methanolic extract.
  (b) CS was detected in the oleolite.
  (c) 12MCA was not detected.
  (d) The total of the abietane-type diterpenes tested in the oil was 8.46% of the amount present in the methanolic extract.

Analysis of the samples showed that there was no identification of RO and 7MER by any method used.

According to Table 10, the descending order of the extractive capacity of the solvents used to soak the leaves of *SR* is MeOH > EtOH > extra virgin olive oil.

Regarding 7MER, it seems that this compound does not exist in the leaves of the tested plants, and is produced when the plant remains in contact with methanol for a certain time. According to Table 12, 7MER does not appear to be initially present in the dry leaves of *SR*, but instead is produced from the decomposition of CA under the influence of methanol with the intermediates CS and RO. In 2 months, 7MER was the dominant compound among the studied abietane-type diterpenes. 7MER has a notable stability in methanol. Indeed, 7MER was still present as the dominant abietane-type diterpene after one year of *SR* leaves maceration in methanol.

The study of the effect of storage time (at 0, 12, 24 and 36 months) of the leaves of Greek sage on the qualitative and quantitative characteristics of the tested abietane-type diterpenes showed that that there is a negative linear correlation between the time period of storage and the total amount of the compounds (Table 11). For all substances, the greatest percentage reduction occurs in the first 12 months of drug storage. Indeed, in the first 12 months, the total quantity of abietane-type diterpenes decreased by 16.51%, at 24 months by 33.61%, while after 36 months, it was reduced by almost half (40.79%) with respect to the start (time 0). The exception was RO, which at time 0 had not been identified, and which appeared at 12 months of storage due to carnosic acid and carnosol decomposition and then decreased with time in the two measurements that followed. 7MER was not identified in any of the four measurements. Moreover, it was observed that at 36 months, the total amount had been reduced by almost half (40.79% reduction).

Regarding previous works that have studied the extraction of salvia species, we would like to note the following:

Sharma et al. (2020) analyzed the decoction of the fresh and dry leaves of *SR* and *SO* using ultra-high-performance liquid chromatography, and electrospray ionization coupled with quadrupole-time of flight mass spectrometry (UHPLC-ESI-QTOF-MS). They boiled the leaves (10 g) of each plant, chopped previously into small pieces, in 200 mL of water at 100–110 °C until the volume became 100 mL. There was not boiling at different times. Their results regarding CA and CS are qualitatively in agreement with ours [23,24].

Two different and contemporary studies, by Zimmermann et al. (2011) and Walch et al. (2011), have determined the polyphenols of aqueous infusions of common sage (adding 150 g boiling water to 1.5 g of leaves or one tea bag for 15 min) using HPLC. Their qualitative and qualitative results regarding CA and RO (as rosmanol isomer) are similar with ours in infusions at 15 min, with the same decreasing order of CA > RO [25,26].

The infusions of Greek sage (by addition of the herb in boiling water, 4 g/100 mL, for 5 min) prepared by Matsingou et al. (2003) present the same qualitatively profile as ours [27]. Oliveira et al. (2016) concluded that 70% ethanol in an ethanol/water mixture was the most efficient for the extraction of CA and CS by rosemary leaves in tandem with our conclusion [28].

Jacotet-Navarro et al. (2018) investigated the solubility of CA in various ethanol/water mixtures by HPLC analysis, and concluded that 100% ethanol was the best solubilizing and also extracting solvent for CA from rosemary leaf. Indeed, the solubility of CA goes to 423.4 g/L in 100% ethanol, starting from 0.1 g/L between 0 and 50% in ethanol. In our opinion, a very important conclusion of this study is that CA is more stable in ethanol than in water, so raising the percent of water in an ethanol/water mixture diminishes the stability of CA. Furthermore, it appears that the degradation rate increases with higher temperatures in the experiments [21].

Nguyen-Kim et al. studied the optimization of the polyphenols’ extraction via maceration from the leaves of *SR*, and concluded that optimal conditions were ethanol 65% (*v*/*v*), 1:7.5 g/mL drug/solvent ratio, temperature of extraction 65 °C, 15 min time of extraction and two cycles of extraction [29].

Li et al. (2019) calculated the extractability of CA and CS after maceration of the dried powdered leaves of *SR* in various vegetable oils, including olive oil, at 40 °C for 3 h. An HPLC system equipped with a photo diode array detector (DAD) was used for the quantitative analysis. Both CA and CS were extracted by olive oil with CA as the dominant compound, as happened in our previous research [30]. Interestingly enough was the result of Li et al. regarding the comparison of the extractive capability between methanol/water (90/10 *v*/*v*) and olive oil regarding CA and CS. According to the authors, in methanolic extract, a minor quantity for CA and a major for CS have been noted. These data are the opposite of ours. We believe that the explanation lies in the method of preparation of the methanolic extract in the work of Li et al. (drug in methanol/water, 90:10 *v*/*v* at the boiling point for 30 min) and the great tendency of CA to degrade under the influence of heating and the presence of the polar solvents (methanol and water). Therefore, we consider that the greater amount of CS in relation to CA is due to the degradation of the latter in polar solvents and under heating, while in the oil, this does not happen because of the great stability of these substances in this solvent, as demonstrated in our present work. In another study, the CA dissolved in virgin olive oil enhanced the stability of the oil to oxidation [31].

Ginsburg et al. (2020) compared via HPLC analysis the ethanol extraction (dried ground leaves of *SR* in ethanol/water, 3:1 *v*/*v* and in a 1:6 ratio *w*/*v* at 40 °C and shaken for 1.5 h) to vegetable oils as high oleic soybean oil, peanut oil and cottonseed oil solution (dry ground leaves, 20–40% *w*/*w*, into vegetable oil between 100–140 °C, on a magnetic stirring for 1–3 h) of rosemary, and found 10–24% more CA in the latter. Hence, they concluded that ethanol/water solvents are less potent to vegetable oils used in extracting CA from *SR* leaves [32]. It is difficult to compare the results of this study to ours. Understanding the differences in study methodologies can indeed make comparisons challenging. Το compare the extractive capability of methanol, ethanol and olive oil with respect to abietane-type diterpenes, we performed the experiments under the same conditions.

## 4. Materials and Methods

### 4.1. Chemicals

The solvents, dichloromethane, ethyl acetate, acetonitrile, ethanol and methanol were of HPLC grade, purchased from Fisher Chemical (Fisher Scientific, Loughborough, Leics, UK). Silica gel 0.040–0.063 mm, TLC (20 × 20 cm) of normal and reverse phase, deuterated methanol (CD_3_OD) and deuterated chloroform (CDCl_3_) were purchased from Merck KGaA (Darmstadt, Germany); olive oil was extra virgin oil “ALTIS” (ELAIS).

### 4.2. Plant Material

Leaves of common sage (*SO*), Greek sage (*SF*) and rosemary (*SR*) were collected in 2019 from J. & A.N. Diomedes Botanic Garden, National and Kapodistrian University of Athens, during the flowering period (July) and dried at room temperature. The dried plant material was ground by a laboratory mill (ZM 200 Retsch, Haan, Germany) using 0.5 mm size hole sieve and stored in darkness at room temperature.

### 4.3. Extracts Preparations

#### 4.3.1. Aqueous Extracts

The drug/solvent ratio was 2:150 *w*/*v* for rosemary and 1.5:150 *w*/*v* for common sage and Greek sage (following the guidelines of the EMA). All experiments were repeated in triplicate.

##### Infusions and Decoctions

Infusions and decoctions were prepared using 4 different times: (i) 2 min, (ii) 5 min, (iii) 10 min and (iv) 15 min, followed by filtering, collection of the filtrates and restoring to the original volume by adding extra volume of water. For the infusions, boiled water was poured onto the plant material and remained in contact for the mentioned time of preparation, while for the decoctions, the plant material remained in contact with continuously boiling water for the mentioned time of preparation.

##### Turbulent Flow Extraction

The extraction was performed using a MRC Heavy duty blender with 1 L stainless steel container and stainless steel blades operating at 22,000 rpm. The plant material and the water were blended for 2 min. Two different experiments were performed: (i) at room temperature (cold turbulent flow extraction) and (ii) with boiled water (hot turbulent flow extraction).

#### 4.3.2. Tinctures

Ground herb, 10 g in 100 mL (or 200 mL) of aqueous alcohol solution, was placed in the ultrasonic water bath for 1 h and then filtered. All tinctures were stored in opaque containers at room temperature and in a dark place until used.

Two different experiments were performed:

(1) With constant drug:solvent ratio 1:10 *w*/*v* and 2 different alcoholic degrees, (1a) 45° and (1b) 70°, in order to compare the two most commonly used alcohol degrees for the preparation of tinctures.
(2) With drug:solvent ratio 1:20 *w*/*v* (as usually mentioned in the literature) with an alcoholic degree of 20°, comparable to liqueurs and wine.

All experiments were repeated in triplicate.

#### 4.3.3. Oleolites

##### Dried Ground Leaves of Rosemary (*SR*)

Two ways for oleolite preparation with selected herbs were used:

- By extracting the herb in vegetable oil by maceration.
- By dissolving an herbal dry extract in the oil.

(i) Maceration at room temperature

Of dried ground leaves, 5 g in 100 g of extra virgin olive oil (D:S = 1:20 *w*/*w*). The airtight closed container was placed in a dark place for 21 days (shaking it once per day). At the end of the extraction, the solid part was separated from the supernatant by decantation and transfusion and filtered through paper filter. The filtrated oil was further analyzed.

(ii) Hot maceration (65 °C “Digestion”)

Two different experiments were performed regarding the leaves-oil ratio: (a) 1:20 and (b) 1:10, keeping the other conditions of the experiment constant. The temperature of the extraction was set at 65 °C for 6 h. The system was stirred every 2 h.

(iii) Dissolution of dry extract in olive oil

Of methanolic *SR* (or *SF*) dry extract, 1 g was transferred to a flask containing 100 g of olive oil, sonicated in an ultrasonic water bath for 30 min and filtered through paper filter. The filtrated oil was further analyzed.

Briefly, the methanolic dry extract was prepared by treating 100 g of the powdered dry leaf of *SR* (or *SF*) with methanol in a 1:10 ratio for 12 h in an opaque recipient. At the appropriate time, the plant material was filtered and the volume of the solution was restored to the original volume (1 L), and 2 L of distilled water was added. The formed precipitate was collected by filtration, dried, and finally powdered to afford the corresponding *SR* (or *SF*) dry extract.

Three measurements were made for each solvent.

All experiments were repeated in triplicate.

#### 4.3.4. Preparation of the Extracts for the Estimation of the Effect of Storage Time of Drug in the Quantity of Abietane-Type Diterpenes

Four measurements were taken, at time 0 (measurement immediately after the drying of the leaves) and after 12, 24 and 36 months. The experiments were carried out with the dried leaves of *SF* previously powdered, extracted in methanol at a ratio of 1:30 *w*/*v* for 30 min in an ultrasonic bath. After that, the extracts were filtered through filter paper, concentrated to dryness and analyzed by ^1^H-NMR (CDCl_3_). Τhe intact leaves were stored in an opaque airtight container in a place protected from light and humidity.

#### 4.3.5. Study on 7MER

The methanolic extracts of the dry leaves of *SR* were prepared and analyzed as described in Section 4.3.4. Τhe dry leaves of *SR* were macerated in methanol for 4 different times: 12 h, 48 h, 7 days and 2 months, at room temperature.

#### 4.3.6. Comparison of the Extractive Capacity of Olive Oil, Ethanol and Methanol

In order to compare the extractive capacity of olive oil, ethanol and methanol, an oleolite, an ethanolic and a methanolic solution of *SR* leaves were prepared under the same conditions: 10.0 g of comminuted dried leaves of *SR* were placed in a 200 mL conical flask, to which 100 mL of solvent (96% ethanol or methanol or olive oil) was added. The extraction was carried out at room temperature in an ultrasonic bath for 60 min. Then the plant material was separated from the liquid part by filtration. The liquid part was collected in a 100 mL round bottom flask and adjusted back to the original volume of 100 mL by adding solvent. Then 10 mL of solution (ethanolic and methanolic) was concentrated under vacuum (40 °C), to obtain a powder which was subjected to 1D ^1^H-NMR spectroscopy (CDCl_3_). Three measurements were made for each solvent.

All experiments were repeated in triplicate.

### 4.4. Nuclear Magnetic Resonance (NMR) Spectroscopy

All chemical shifts were measured with reference to the internal standard, 3-trimethylsilyl-2,2′,3′,3′-tetradeuteropropionic acid (TMSP-d_4_) (δH = 0.000 ppm), of known concentration (0.3 mmol L-1). All experiments were performed on a Bruker DRX 500 MHz NMR spectrometer (Bruker Biospin, Rheinstetten, Germany) operating at NMR frequency of 500.13 MHz for ^1^H and 125.77 MHz for ^13^C NMR. All ^1^H (500 MHz) and ^13^C NMR (125 MHz) spectra were recorded with chemical shifts (ppm) and coupling constants (J) in hertz (Hz). CDCl_3_ was used in NMR analysis due to the sensibility of the abietane-type tested metabolites in polar solvents [32,33]. CD_3_OD was used only for the pure compounds in order to confirm their identification.

### 4.5. Isolation of Secondary Metabolites from the Studied Plants

The studied secondary metabolites of abietane-type diterpenes, carnosic acid (**2**), carnosol (**3**) and 7-*O*-methyl-*epi*-rosmanol (**4**), were isolated and structurally determined as described previously [18]. The isolation process for 12-*O*-methyl-carnosic acid (**1**) and rosmanol (**5**) is described below.

#### 4.5.1. Isolation of 12MCA (**1**)

Although 12MCA was present in the three studied plants, the dried leaves of *Salvia microphylla* Kunth were used as an alternative richer source. Of powdered leaves, 15 g was extracted with 200 mL MeOH for 2 h in an ultrasonic water bath. The extract was evaporated in vacuo to dryness yielding 2.1751 g [SMe] of deep green residue. [SMe] was treated with a mixture of water: methanol 2:1, (150 mL), for 5 min in ultrasonic water bath, then filtered and further treated with acetone (25 mL) and concentrated to give 1.9071 g of [SM]. The NMR spectrum of the extract showed the presence of 12MCA as the major abietane-type diterpene (Figure S5). Of [SΜ], 1.23 g was further subjected to column chromatography on silica gel (51 × 3 cm; silica gel 20.0 g) and eluted with cyclohexane (CHx)-ethyl acetate (EtOAc) (gradient 100% of CHx–CHx: EtOAc = 85:15% *v*/*v*) to provide 69 fractions (1–5, 40 mL; 6–69, 15 mL). Fractions 46–50 (35.1 mg) contained 12MCA identified by NMR [17]. The NMR spectra of 12MCA in CDCl_3_ and CD_3_OD are given in Figures S1 and S2 and Tables S1 and S2, respectively.

#### 4.5.2. Isolation of Rosmanol (RO) (**5**)

Of ground leaves of *S. fruticosa*, 3 g was used to prepare decoctions with 300 mL of water boiling for 10 min. The decoction was filtered and the filtrate further extracted with 150 mL CH_2_Cl_2_. The organic phase was then concentrated [SF-d] (42.7 mg). Of [SF-d], 23.7 mg was subjected to normal phase pTLC (CH_2_Cl_2_: CH_3_OH = 97:3 *v*/*v*) to afford rosmanol (1.9 mg) identified by 1D and 2D NMR spectroscopy and comparison with bibliographic data [34,35]. The NMR spectra of RO in CDCl_3_ and CD_3_OD are given in Figures S3 and S4 and Tables S3 and S4, respectively. An example of the ^1^H-NMR spectrum of the *S. fruticosa* extract showing the peaks used for quantitation is given in the Supplementary Material (Figure S6).

### 4.6. Development of Analytical Method

#### 4.6.1. Aqueous Extracts (Infusions, Decoctions, Turbulent Flow Extracts)

Each aqueous extract at room temperature was placed into a separatory funnel with an equal volume of CH_2_Cl_2_. After shaking and allowing to stand for 20 min, the organic phase was collected and concentrated to dryness followed by 1D ^1^H-qNMR (CDCl_3_). Examples of NMR spectra of the decoctions of the three studied plants in comparison with the spectra of the pure studied metabolites are provided in Figures S7 and S8.

#### 4.6.2. Tinctures

(1) Samples of tinctures of 45° and 70°: 10 mL of the tincture was concentrated to dryness and 1D ^1^H-qNMR spectra were obtained at 298 K in CDCl_3_.

(2) Samples of tinctures of 20°: 20 mL of the tincture was placed in a separatory funnel together with an equal volume of CH_2_Cl_2_. After shaking and allowing to stand for 20 min, the organic phase was concentrated to dryness followed by 1D ^1^H-qNMR at 298 K in CDCl_3_.

#### 4.6.3. Oleolites

The method for the analysis of oleolites was based on a previously described method for olive oil’s polyphenols [36]: 5 g of oleolite together with 20 mL cyclohexane (CHx) was shaken vigorously for 1 min. Then 25 mL of acetonitrile (ACN) was added, resulting in liquid–liquid phase separation (polar of ACN and non-polar with CHx and olive oil). The whole mixture was then centrifugated (4000 rpm for 5 min). Finally, 25 mL of ACN solution was concentrated and 1D ^1^H-qNMR acquisition was carried out at 298K in CDCl_3_.

### 4.7. Statistical Analysis

Depending on treatments data, analysis of variance and Duncan’s multiple range test, or paired samples *T*-Test or *T*-test, at *p* ≤ 0.05, were performed to assess the extraction method for the targeted bioactive compounds of abietane-type diterpenes present in the following treatments: (i) infusions, (prepared for 2, 5, 10 and 15 min), (ii) decoctions (in 2, 5, 10 and 15 min), (iii) turbulent flow extractions (at room temperature and at 100 °C), (iv) tinctures (in different ethanol volumes 20% and 70%) and (v) oleolites (prepared at room temperature and at 65 °C). All statistical analysis was performed using SPSS v.20 software for Windows (IBM SPSS Statistics 2011, IBM Corp., Armonk, NY, USA).

Calibration curves statistical analysis was carried out using Microsoft Excel Analysis Toolpack. Regression and correlation analyses were made by using the regression analysis module of Microsoft Excel 2016 software version 2312 (Microsoft, Washington, DC, USA).

## 5. Conclusions

Several traditionally used herbal preparations for oral use (infusions, decoctions, tinctures and oleolites) and for first-time turbulent flow extraction, from *S. fruticosa, S. officinalis* and *S. rosmarinus*, were studied regarding the content of the abietane-type diterpenes. The variations in compound concentrations observed with each extraction method may be attributed to the instability of these compounds in polar solvents and high temperatures during the extraction process. These results underscore the importance of conducting comprehensive studies in the future for other commonly used herbal drugs regarding the effect of the extraction/preparation on the concentration of specific active metabolites.

In general, for all three studied plants, the method of decoction was found more efficient than the infusion and hot turbulent flow extraction was almost equal to decoction. It was also found that the tincture is not a suitable formulation for the abietane-type diterpenes due to their instability in a hydroalcoholic solvent at 70°. In addition, the olive oil extract of rosemary was investigated and it showed that the metabolites CA, CS and 12MCA were detected in the extra virgin olive oil and remained stable in this solvent, which is particularly important for these valuable metabolites, while any edible oil, and especially extra virgin olive oil in our case, is a totally non-toxic solvent capable of applying the extract directly and safely for oral use and to the final food products. The established ^1^H-qNMR method offers a fast, simple, reliable and accurate method for the quantitative and qualitative analysis of the selected abietane-type diterpenes, in the complex extracts of the tested Lamiaceae herbs in a direct way with high precision and without sample deterioration.

## Figures and Tables

**Table 1:** Calibration curves, LOD and LOQ values.

Compounds	y = ax + b	R^2^	LOD	LOQ
12MCA	y = 0.4696x − 0.0021	0.9999	0.04	0.1
CA	y = 0.4812x − 0.0067	0.9999	0.04	0.1
CS	y = 0.4565x − 0.0043	0.9999	0.04	0.1
7MER	y = 0.4917x − 0.0025	0.9999	0.04	0.1
RO	y = 0.4736x − 0.003	0.9999	0.04	0.1

**Table 2:** Quantities of the studied metabolites obtained in aqueous extracts (expressed as mg ± SD/g dry leaves) of rosemary (*SR*).

Extract	Compounds	2 Min	5 Min	10 Min	15 Min
Infusions	12MCA	tr	tr	tr	tr
	CA	tr	tr	tr	0.18 ± 0.05
	CS	tr	tr	tr	tr
	7MER	nd	nd	nd	nd
	RO	0.25 ± 0.03 ^b^	0.32 ± 0.08 ^ab^	0.42 ± 0.07 ^a^	0.21 ± 0.03 ^b^
	Total	0.25 ± 0.03 ^b^	0.32 ± 0.08 ^ab^	0.42 ± 0.07 ^a^	0.39 ± 0.05 ^a^
Decoctions	12MCA	tr	tr	0.12 ± 0.04 ^a^	0.11 ± 0.04 ^a^
	CA	tr	tr	0.16 ± 0.07 ^b^	0.29 ± 0.08 ^a^
	CS	nd	nd	tr	tr
	7MER	nd	nd	nd	nd
	RO	0.30 ± 0.04 ^b^	0.38 ± 0.05 ^ab^	0.40 ± 0.06 ^a^	0.40 ± 0.02 ^a^
	Total	0.30 ± 0.04 ^c^	0.38 ± 0.05 ^c^	0.68 ± 0.04 ^b^	0.80 ± 0.09 ^a^
Turbulent flow extractions at RT	12MCA	nd	nm	nm	nm
	CA	nd	nm	nm	nm
	CS	tr	nm	nm	nm
	7MER	nd	nm	nm	nm
	RO	nd	nm	nm	nm
	Total	tr	nm	nm	nm
Turbulent flow extraction under heating	12MCA	0.12 ± 0.01	nm	nm	nm
	CA	0.32 ± 0.03	nm	nm	nm
	CS	0.17 ± 0.02	nm	nm	nm
	7MER	nd	nm	nm	nm
	RO	0.23 ± 0.03	nm	nm	nm
	Total	0.84 ± 0.04	nm	nm	nm

Total of the abietane-type diterpenes examined as a sum of the sub averages. tr: trace, (≥LOD, <LOQ), nd: not detected, (<LOD), nm: not measured, RT: room temperature. Means followed by the same letter in the same line do not differ statistically at *p* = 0.05.

**Table 3:** Quantities of the studied metabolites obtained in aqueous extracts (expressed as mg ± SD/g dry leaves) of Greek sage (*SF*).

Extract	Compounds	2 Min	5 Min	10 Min	15 Min
Infusions	12MCA	0.10 ± 0.01 ^b^	0.11 ± 0.01 ^b^	0.13 ± 0.01 ^a^	tr
	CA	0.13 ± 0.01 ^d^	0.37 ± 0.08 ^c^	0.56 ± 0.06 ^b^	0.75 ± 0.09 ^a^
	CS	tr	tr	0.13 ± 0.01 ^b^	0.19 ± 0.01 ^a^
	7MER	nd	nd	nd	nd
	RO	0.52 ± 0.04 ^a^	0.42 ± 0.03 ^b^	0.34 ± 0.02 ^c^	0.34 ± 0.02 ^c^
	Total	0.75 ± 0.06 ^b^	0.90 ± 0.11 ^b^	1.16 ± 0.08 ^a^	1.28 ± 0.10 ^a^
Decoctions	12MCA	0.20 ± 0.03 ^b^	0.32 ± 0.05 ^a^	0.34 ± 0.03 ^a^	0.33 ± 0.07 ^a^
	CA	0.86 ± 0.09 ^b^	1.62 ± 0.18 ^a^	1.38 ± 0.12 ^a^	1.33 ± 0.38 ^a^
	CS	0.14 ± 0.06 ^b^	0.26 ± 0.10 ^a^	0.25 ± 0.06 ^a^	0.17 ± 0.06 ^b^
	7MER	nd	nd	nd	Nd
	RO	0.96 ± 0.01 ^b^	1.12 ± 0.19 ^ab^	1.24 ± 0.03 ^a^	1.00 ± 0.08 ^b^
	Total	2.16 ± 0.10 ^b^	3.32 ± 0.37 ^a^	3.21 ± 0.20 ^a^	2.83 ± 0.41 ^a^
Turbulent flow extractions at RT	12MCA	tr	nm	nm	nm
	CA	tr	nm	nm	nm
	CS	0.19 ± 0.10	nm	nm	nm
	7MER	nd	nm	nm	nm
	RO	nd	nm	nm	nm
	Total	0.19 ± 0.10	nm	nm	nm
Turbulent flow extraction under heating	12MCA	0.17 ± 0.04	nm	nm	nm
	CA	1.12 ± 0.44	nm	nm	nm
	CS	0.24 ± 0.03	nm	nm	nm
	7MER	nd	nm	nm	nm
	RO	0.53 ± 0.18	nm	nm	nm
	Total	2.06 ± 0.61	nm	nm	nm

Total of the abietane-type diterpenes examined as a sum of the sub averages. tr: trace, (≥LOD, <LOQ), nd: not detected, (<LOD), nm: not measured, RT: room temperature. Means followed by the same letter in the same line do not differ statistically at *p* = 0.05.

**Table 4:** Quantities of the studied metabolites obtained in aqueous extracts (expressed as mg ± SD/g dry leaves) of common sage (*SO*).

Extract	Compounds	2 Min	5 Min	10 Min	15 Min
Infusions	12MCA	0.31 ± 0.02 ^b^	0.31 ± 0.02 ^b^	0.39 ± 0.03 ^a^	0.31 ± 0.04 ^b^
	CA	0.48 ± 0.03 ^b^	0.50 ± 0.02 ^b^	0.62 ± 0.04 ^a^	0.64 ± 0.02 ^a^
	CS	0.13 ± 0.01 ^b^	0.13 ± 0.01 ^b^	0.15 ± 0.01 ^b^	0.18 ± 0.02 ^a^
	7MER	nd	nd	nd	nd
	RO	0.14 ± 0.02 ^b^	0.14 ± 0.02 ^b^	0.24 ± 0.08 ^a^	0.16 ± 0.01 ^b^
	Total	1.06 ± 0.05 ^b^	1.08 ± 0.03 ^b^	1.40 ± 0.14 ^a^	1.29 ± 0.08 ^a^
Decoctions	12MCA	0.84 ± 0.02 ^b^	1.02 ± 0.03 ^a^	0.95 ± 0.05 ^ab^	0.74 ± 0.03 ^c^
	CA	1.25 ± 0.02 ^b^	1.52 ± 0.13 ^a^	0.81 ± 0.12 ^c^	0.60 ± 0.02 ^c^
	CS	tr	0.11 ± 0.02 ^b^	0.17 ± 0.03 ^a^	0.12 ± 0.01 ^b^
	7MER	nd	nd	nd	nd
	RO	0.65 ± 0.04 ^b^	0.74 ± 0.09 ^ab^	0.85 ± 0.05 ^a^	0.41 ± 0.07 ^c^
	Total	2.74 ± 0.02 ^b^	3.39 ± 0.09 ^a^	2.78 ± 0.09 ^b^	1.87 ± 0.07 ^c^
Turbulent flow extractions at RT	12MCA	0.22 ± 0.02	nm	nm	nm
	CA	0.11 ± 0.01	nm	nm	nm
	CS	0.21 ± 0.02	nm	nm	nm
	7MER	nd	nm	nm	nm
	RO	nd	nm	nm	nm
	Total	0.54 ± 0.02	nm	nm	nm
Turbulent flow extraction under heating	12MCA	0.95 ± 0.04	nm	nm	nm
	CA	1.59 ± 0.16	nm	nm	nm
	CS	0.30 ± 0.03	nm	nm	nm
	7MER	nd	nm	nm	nm
	RO	0.11 ± 0.03	nm	nm	nm
	Total	2.95 ± 0.14	nm	nm	nm

Total of the abietane-type diterpenes examined as a sum of the sub averages. tr: trace, (≥LOD, <LOQ), nd: not detected, (<LOD), nm: not measured, RT: room temperature. Means followed by the same letter in the same line do not differ statistically at *p* = 0.05.

**Table 5:** Concentrations of the studied metabolites in the obtained tinctures of rosemary (*SR*) prepared with different ethanol percentages and drug to solvent ratio and expressed as mg ± SD/100 mL.

Compounds	1:10, (10:100 *w*/*v*), 70% EtOH	1:10, (10:100 *w*/*v*), 45% EtOH	1:20, (5:100 *w*/*v*), 20% EtOH
12MCA	2.61 ± 0.20	tr	tr
CA	11.16 ± 1.03	tr	tr
CS	3.24 ± 0.27 ^a^	2.13 ± 0.14 ^b^	tr
7MER	nd	nd	nd
RO	tr	tr	2.36 ± 0.11
T ^1^	17.01 ± 1.20 ^a^	2.13 ± 0.14 ^b^	2.36 ± 0.11 ^b^

^1^ Total abietane-type diterpenes tested as sum of individual averages, tr: trace, nd: not detected. Means followed by the same letter in the same line do not differ statistically at *p* = 0.05.

**Table 6:** Concentrations of the studied metabolites in the obtained tinctures (ethanol 70%) of rosemary (*SR*) at different storage times (1, 7 and 14 days) expressed as mg ± SD/100 mL. tr: trace, nd: not detected.

Compounds	1 Day	7 Days	14 Days
12MCA	2.61 ± 0.20 ^a^	1.46 ± 0.09 ^b^	tr
CA	11.16 ± 1.03 ^a^	3.09 ± 0.11 ^b^	tr
CS	3.24 ± 0.27 ^b^	6.75 ± 0.54 ^a^	1.29 ± 0.08 ^c^
7MER	nd	nd	nd
RO	tr	tr	tr
T ^1^	17.01 ± 1.20 ^a^	11.31 ± 0.61 ^b^	1.29 ± 0.08 ^c^

^1^ Total abietane-type diterpenes tested as sum of individual averages. Means followed by the same letter in the same line do not differ statistically at *p* = 0.05.

**Table 7:** Concentration of the studied metabolites in oleolites of rosemary (*SR*) prepared by maceration in heating (“digestion”) and at room temperature. Results expressed as mg ± SD/100 g of oleolite.

Compounds	1:20 *w*/*w* 65 °C, 6 h	1:10 *w*/*w* 65 °C, 6 h	1:20 *w*/*w* 25 °C, 21 days
12MCA	tr	tr	tr
CA	tr	5.37 ± 0.60 ^a^	2.55 ± 0.23 ^b^
CS	tr	tr	tr
7MER	nd	nd	nd
RO	nd	nd	nd
T ^1^	tr	5.37 ± 0.60 ^a^	2.55 ± 0.23 ^b^

^1^ Total of the abietane-type diterpenes tested as a sum of the sub averages, tr: trace, nd: not detected. Means followed by the same letter in the same line do not differ statistically at *p* = 0.05.

**Table 8:** Concentration of the studied metabolites in oleolites of rosemary and Greek sage expressed as mg ± SD/100 g of oleolite and the corresponding methanolic extracts expressed as mg ± SD/g of dry extract.

Compounds	Methanolic Extract Rosemary (*SR*)	Oleolite Rosemary (*SR*)	Methanolic Extract Greek Sage (*SF*)	Oleolite Greek Sage (*SF*)
12MCA	15.51 ± 2.23	tr	7.13 ± 0.76	nd
CA	59.07 ± 5.35 ^a^	7.18 ± 0.68 ^b^	55.43 ± 5.88 ^a^	6.97 ± 0.68 ^b^
CS	21.51 ± 2.76	tr	19.82 ± 1.92	tr
7MER	nd	nd	nd	nd
RO	nd	nd	nd	nd
T ^1^	96.09 ± 6.89 ^a^	7.18 ± 0.68 ^b^	82.38 ± 6.88 ^a^	6.97 ± 0.68 ^b^

^1^ Total of the abietane-type diterpenes tested as a sum of the sub averages, tr: trace, nd: not detected. Means followed by the same letter in the same line do not differ statistically at *p* = 0.05.

**Table 9:** Stability of the examined metabolites of rosemary (*SR*) in the oleolite depending on storage time (mg ± SD in 100 g of oleolite).

Compounds	0 Time	2 Months
12MCA	tr	tr
CA	2.55 ± 0.23 ^a^	2.44 ± 0.31 ^a^
CS	tr	tr
7MER	nd	nd
RO	nd	nd
T ^1^	2.55 ± 0.23 ^a^	2.44 ± 0.31 ^a^

^1^ Total of the abietane-type diterpenes tested as a sum of the sub averages, tr: trace, nd: not detected. Means followed by the same letter in the same line do not differ statistically at *p* = 0.05.

**Table 10:** Comparison of the extractive performance of olive oil, ethanol and methanol solvents with rosemary (*SR*) leaves as raw material. Results are expressed as mg ± SD of substances in 100 mL of preparation and correspond to 3 measurements.

Compounds	Oleolite	Ethanol	Methanol
12MCA	tr	16.11 ± 2.35 ^a^	20.3 ± 6.65 ^a^
CA	tr	46.81 ± 5.43 ^b^	69.93 ± 8.14 ^a^
CS	tr	8.60 ± 1.00 ^a^	10.68 ± 1.34 ^a^

tr: trace. Means followed by the same letter in the same line do not differ statistically at *p* = 0.05.

**Table 11:** Average of 2 measurements (mg/g dry leaves) of abietane-type diterpenes tested depending on storage time for Greek sage (*SF*).

Compounds	0 Months (Start)	12 Months	24 Months	36 Months
12MCA	1.02	0.85	0.73	0.64
CA	6.52	5.24	4.16	3.68
CS	0.82	0.69	0.53	0.49
7MER	nd	nd	nd	nd
RO	nd	0.20	0.13	0.14
T ^1^	8.36	6.98	5.55	4.95

^1^ Total of the abietane-type diterpenes tested as a sum of the sub averages, nd: not detected.

**Table 12:** Maceration of rosemary *(**SR**)* dry leaves in methanol. Results expressed as mg ± SD/g dry methanolic extract (3 measurements).

Compound	12 h	48 h	7 Days	2 Months
7MER	nd	nd	tr	32.3 ± 2.9

tr: trace; (≥LOD, <LOQ), nd: not detected; <LOD.

## Data Availability

The data presented in this study are available on request from the corresponding author.

## Supplementary Materials

The following supporting information can be downloaded at: https://www.mdpi.com/article/10.3390/molecules29030625/s1, Figure S1: ^1^H-NMR spectrum of 12-*O*-methylcarnosic acid (12MCA) in CDCl_3_; Figure S2: ^1^H-NMR spectrum of 12-*O*-methylcarnosic acid (12MCA) in CD_3_OD; Figure S3: ^1^H-NMR spectrum of Rosmanol (RO) in CDCl_3_; Figure S4: ^1^H-NMR spectrum of Rosmanol (RO) in CD_3_OD; Figure S5: ^1^H-NMR spectrum in CDCl_3_ of methanolic extract of *Salvia microphylla* Kunth.; Figure S6: ^1^H-NMR spectrum in CDCl_3_ of *Salvia fruticosa* Mill. decoction in 5 min; Figures S7 and S8: ^1^H-NMR spectra of decoctions of SR, SO, SF in comparison with the pure studied metabolites. Table S1: ^1^H-NMR, ^13^C-NMR data of 12MCA in CDCl_3_; Table S2: ^1^H-NMR of 12MCA in CD_3_OD; Table S3: ^1^H-NMR, ^13^C-NMR data of RO in CDCl_3_; Table S4: ^1^H-NMR, ^13^C-NMR data of RO in CD_3_OD.

## Abbreviations

12MCA	12-*O*-methyl-carnosic acid
^1^H-qNMR	Quantitative Proton Nuclear Magnetic Resonance Spectroscopy
7MER	7-*O*-methyl-*epi*-rosmanol
AhR	aryl hydrocarbon receptor also called dioxin receptor
CA	carnosic acid
CS	carnosol
FICZ	6-formylindolo [3,2-*b*]carbazole
IND	indirubin
ΝΜR	Nuclear Magnetic Resonance Spectroscopy
PZ	pityriazepin
RO	rosmanol
SF	Greek sage, *Salvia fruticosa* Mill.
SO	Common sage, *Salvia officinalis* L.
SR	Rosemary sage, *Salvia rosmarinus* Spenn syn. *Rosmarinus officinalis* L.
TCDD	2,3,7,8-tetrachlorodibenzo-*p*-dioxin
