# Supplementary material for: Extraction Optimization and Qualitative/Quantitative Determination of Bioactive Abietane-Type Diterpenes from Three Salvia Species (Common Sage, Greek Sage and Rosemary) by 1H-qNMR
Source: Molecules. 2024 Jan 28;29(3):625. doi: 10.3390/molecules29030625 (PMC10856435; doi:10.3390/molecules29030625)
Supplement: Supplementary file 1 [file molecules-29-00625-s001.zip › molecules-2777763-supplementary.pdf]

SUPPLEMENTARY MATERIAL

Extraction optimization and Qualitative/ Quantitative determination of bioactive abietane-type diterpenes from three *Salvia* Species (common sage, Greek sage and rosemary) by ^{1}H-qNMR

Panagiotis Kallimanis [1], Prokopios Magiatis [1, *], Angeliki Panagiotopoulou [2], Ioanna Chinou [1,*]

1 Laboratory of Pharmacognosy & Chemistry of Natural Products, Department of Pharmacy, National & Kapodistrian University of Athens, University Campus 157 71 Zografou, Greece
2 Institute of Biosciences & Applications, National Centre for Scientific Research "Demokritos", 15310 Agia Paraskevi Attikis, Greece
* Correspondence: P.M. Tel.: +30 2107274052; magiatis@pharm.uoa.gr. I.C.: ichinou@pharm.uoa.gr

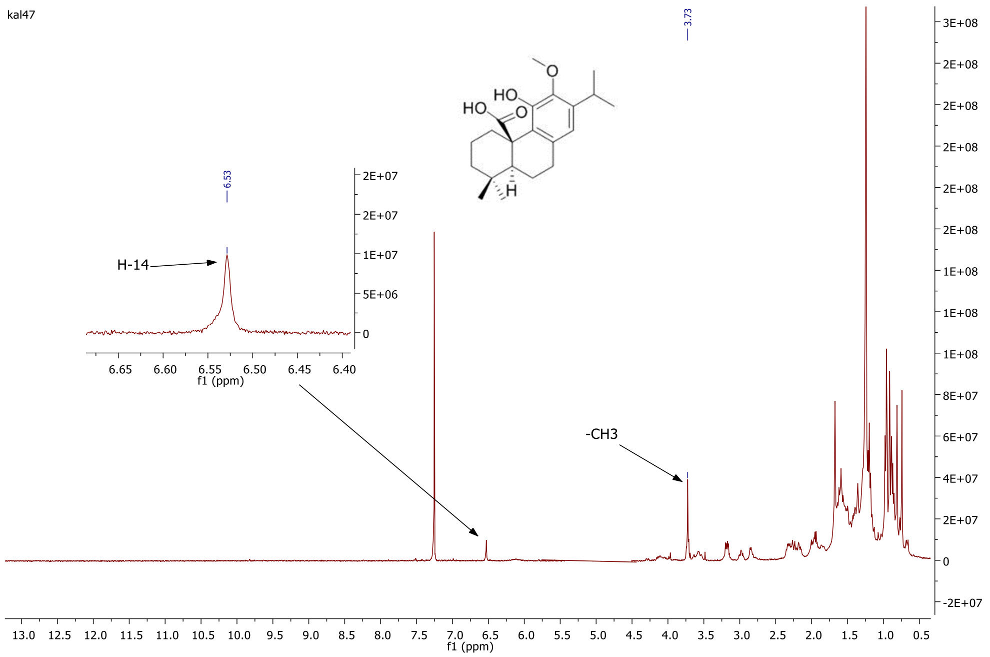

Figure S1 ^{1}H-NMR spectrum of 12-O-methylcarnosic acid (12MCA) in $CDCl_3$

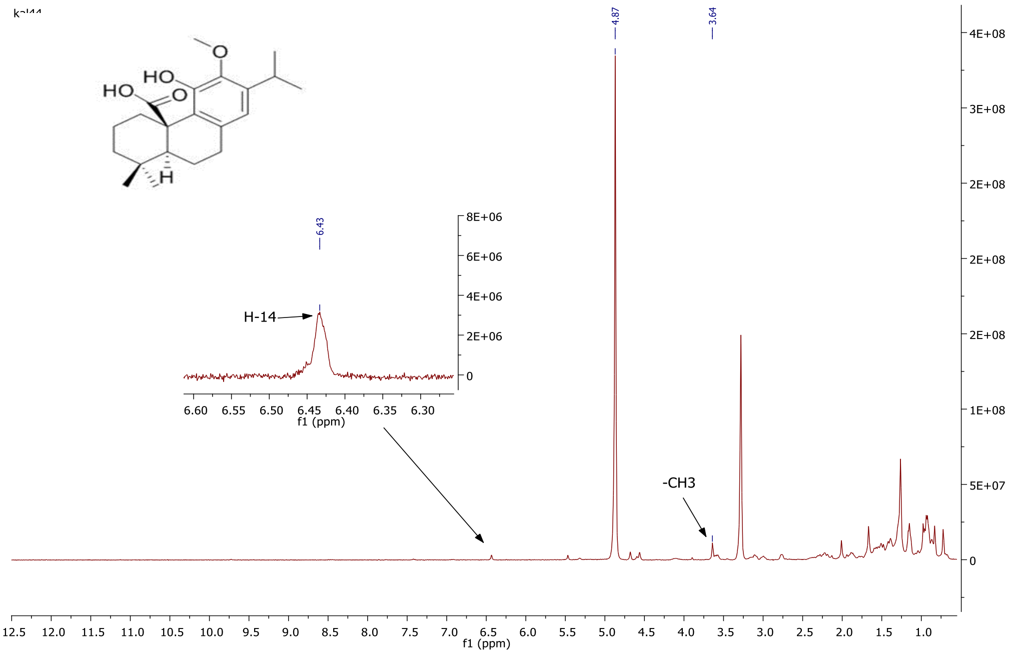

Figure S2 ^{1}H-NMR spectrum of 12-O-methylcarnosic acid (12MCA) in CD_3OD

Table S1 ^{1}H-NMR, ^{13}C-NMR data of 12MCA in $CDCl_3$.

No	δ ^{1}H (ppm)	Integration	Multiplicity	J (Hz)	δ ^{13}C (ppm)
1	α 1.16<br>β 3.57	1<br>1	m<br>m	 -	33.8
2	α 2.16<br>β 1.56	1<br>1	m<br>m	-<br>-	19.6
3	α 1.53<br>β 1.30	1<br>1	m<br>m	-	41.7
4	-	-	-	-	34.1
5	1.59	1	m	-	59.8
6	α 1.85<br>β 2.25	1<br>1	m<br>m	-<br>-	19.7
7	α 2.84<br>β 0.96	1<br>1	m<br>d	-<br>2.5	34.3
8	-	-	-	-	134.6
9	-	-	-	-	125.3
10	-	-	-	-	47.8
11, -OH	6.13	1	br s		145.9
12	-	-	-	-	147.1
13	-	-	-	-	139.7
14	6.53	1	s	-	117.5
15	3.18	1	sept	6.7	26.7
16	1.22	3	m	-	23.5
17	1.19	3	m	-	23.9
18	0.98	3	s		33.3
19	0.87	3	s		18.9
20	-	-	-	-	178.9
$-CH_3$	3.73	3	s		54.8

Table S2 ^{1}H-NMR of 12MCA in CD_3OD.

No	δ ^{1}H (ppm)	Integration	Multiplicity	J (Hz)
1	α 1.07<br>β 3.59	1<br>1	ddd<br>m	12.9/ 12.2/ 4.4
2	α 2.22<br>β 1.53	1<br>1	m<br>m	
3	α 1.53<br>β 1.32	1<br>1	m<br>m	
4	-	-	-	-
5	1.51	1	m	
6	α 1.77<br>β 2.22	1<br>1	brd<br>m	12
7	α 2.76<br>β 2.76	1<br>1	m<br>m	
8	-	-	-	-
9	-	-	-	-
10	-	-	-	-
11	-	-	-	-
12	-	-	-	-
13	-	-	-	-
14	6.43	1	s	
15	3.13	1	m	
16	1.13	3	d	7.1
17	1.15	3	d	7.3
18	0.98	3	s	
19	0.83	3	s	
20	-	-	-	-
$-CH_3$	3.64	3	s	

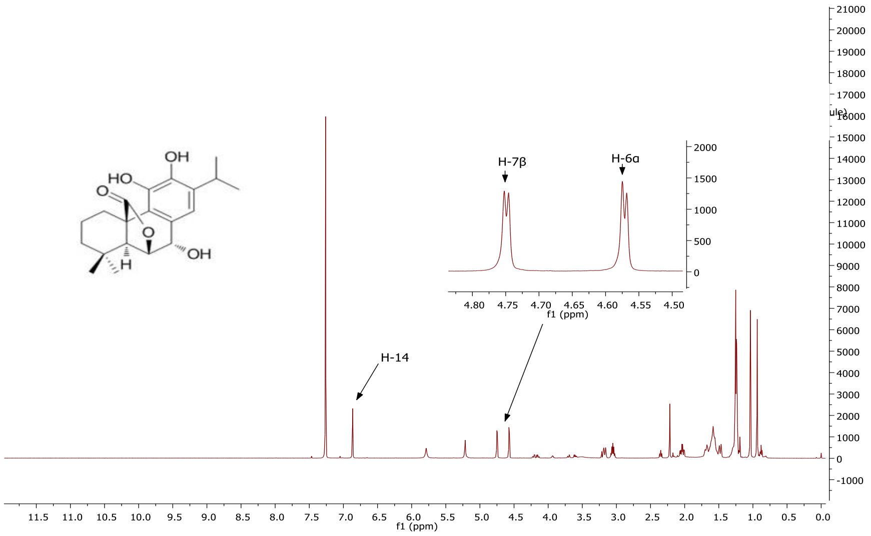

Figure S3 ^{1}H-NMR spectrum of Rosmanol (RO) in $CDCl_3$

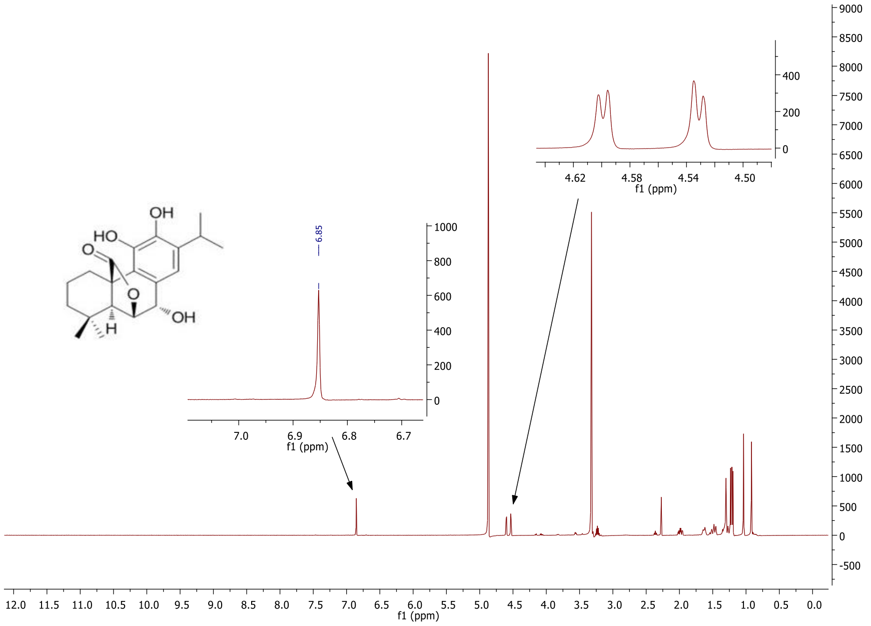

Figure S4 ^{1}H-NMR spectrum of Rosmanol (RO) in CD_3OD

Table S3 ^{1}H-NMR, ^{13}C-NMR data of RO in $CDCl_3$

Position	δ ^{1}H (ppm)	Integration	Multiplicity	J (Hz)	δ ^{13}C (ppm)
1	α 2.03<br>β 3.17	1<br>1	td<br>brd	 13.8	28.5
2	α 1.52<br>β 1.45	1<br>1	m<br>m		19.7
3	α 1.17<br>β 1.43	1<br>1	m<br>m		38.4
4	-	-	-	-	31.9
5	2.2	1	s		55.6
6	4.57	1	d	3.3	70.9
7	4.75	1	d	3.3	80.3
8	-	-	-	-	131.2
9	-	-	-	-	136.8
10	-	-	-	-	48.8
11	-	-	-	-	144.3
12	-	-	-	-	145.3
13	-	-	-	-	125.4
14	6.86	1	s		118.9
15	3.05	1	sept	7	27.7
16	1.23	3	d	4.5	22.2
17	1.25	3	d	4	22.9
18	1.04	3	s		31.6
19	0.94	3	s		23.3
20	-	-	-	-	178.9

Table S4 ^{1}H-NMR, ^{13}C-NMR data of RO in CD_3OD.

Position	δ ^{1}H (ppm)	Integration	Multiplicity	J (Hz)	δ ^{13}C (ppm)
1	α 1.98<br>β 1.48	1<br>1	td<br>m	14.4/ 5.2	28.7
2	α 1.45<br>β 1.61	1<br>1	m<br>m		20.5
3	α 1.25<br>β 3.32	1<br>1	td<br>m	13.2/ 3	39.9
4	-	-	-	-	32.3
5	2.27	1	s		52.1
6	4.53	1	d	3.2	80.6
7	4.60	1	d	3.3	69.5
8	-	-	-	-	129.8
9	-	-	-	-	125.6
10	-	-	-	-	48.9
11	-	-	-	-	145.6
12	-	-	-	-	143.9
13	-	-	-	-	138.2
14	6.85	1	s		120.7
15	3.23	1	sept	6.9	27.9
16	1.22	3	d	6.9	22.8
17	1.20	3	d	6.9	23.3
18	1.03	3	s		32.1
19	0.92	3	s		22.6
20	-	-	-	-	181.2

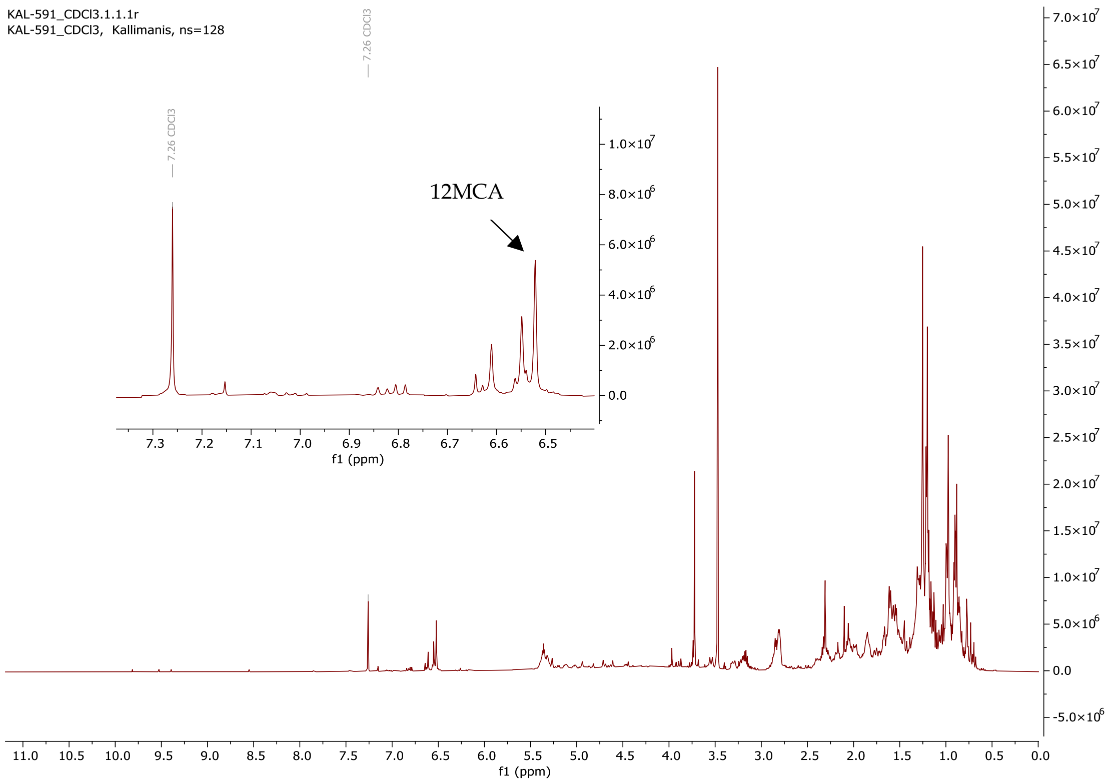

Figure S5 ^{1}H-NMR spectrum in $CDCl_3$ of methanolic extract of *Salvia microphylla* Kunth.

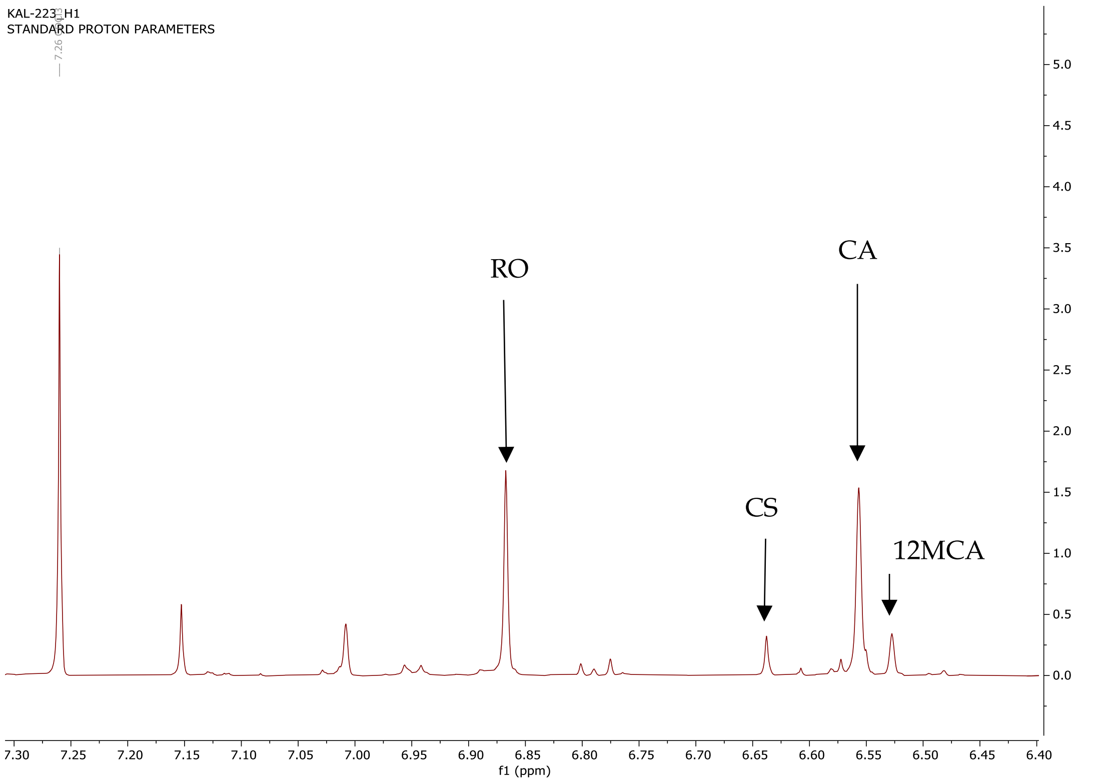

Figure S6 ^{1}H-NMR spectrum in $CDCl_3$ of *Salvia fruticosa* Mill. decoction in 5 min (CA: carnosic acid, CS: carnosol, 12MCA: 12-O-methylcarnosic acid, RO: rosmanol).

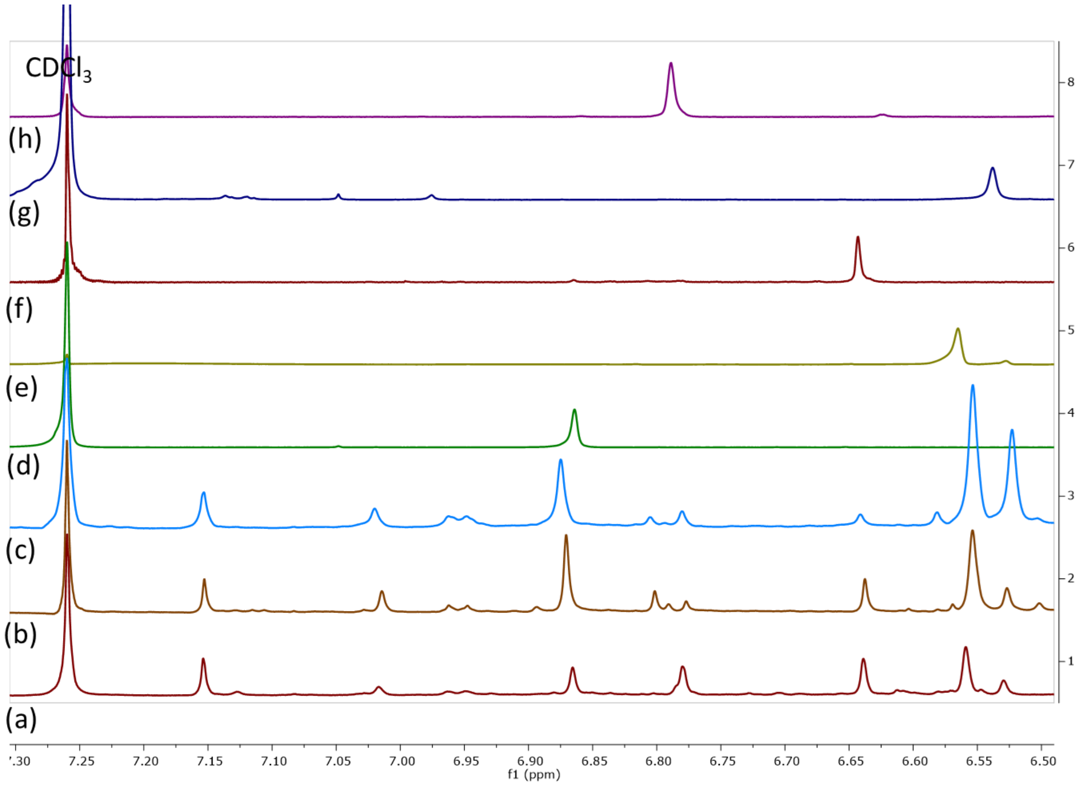

Figure S7 ^{1}H-NMR spectra of aqueous extracts (decoction in 5 min) of *Salvia rosmarinus* Spenn. (a), *Salvia fruticosa* Mill. (b) and *Salvia officinalis* L. (c), Rosmanol (d), Carnosic acid (e), Carnosol (f), 12-O-methylcarnosic acid (g) and 7-methoxy epirosmanol (h), in $CDCl_3$ (set at 7.26 ppm, range 7.3 -6.5 ppm).

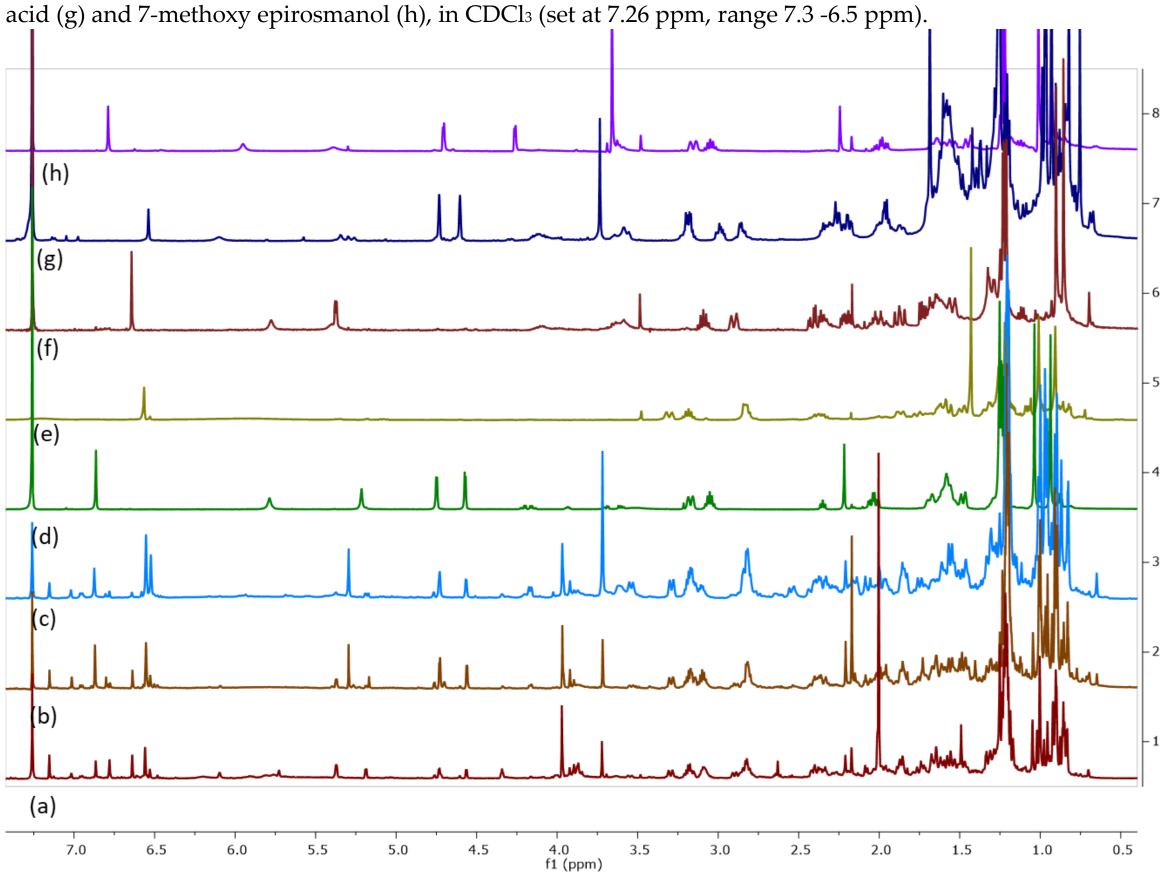

Figure S8 ^{1}H-NMR spectra of of aqueous extracts (decoction in 5 min) of *Salvia rosmarinus* Spenn. (a), *Salvia fruticosa* Mill. (b) and *Salvia officinalis* L. (c), Rosmanol (d), Carnosic acid (e), Carnosol (f), 12-O-methylcarnosic acid (g) and 7-methoxy epirosmanol (h), in $CDCl_3$ (set at 7.26 ppm, range 7.4 -0.5 ppm).
